# Task-Evoked Whole-Brain Functional Connectivity Predicts Post-Training Go-Choice Bias After Food Go/No-Go Training

**DOI:** 10.3390/bs16101799

**Published:** 2026-10-02

**Authors:** Yingkai Yang

**Affiliations:** Faculty of Psychology, Southwest University, Chongqing 400715, China; yangyk0725@swu.edu.cn; Tel.: +86-(0)23-68252376

**Keywords:** food go/no-go training, post-training Go-choice bias, task-evoked functional connectivity, connectome-based predictive modeling

## Abstract

Individual responses to food go/no-go training vary substantially, but the task-evoked network correlates of this variability remain unclear. This study tested whether large-scale functional connectivity during food choice predicts individual differences in post-training Go-choice bias. A behavioral sample of 172 young women completed food go/no-go training in which Go and No-Go cues were paired with value-matched food items, followed by an in-scanner binary food-choice task; usable task-based fMRI data were available for 162 participants. Task-modulated connectivity among 268 brain regions was estimated using generalized psychophysiological interaction analysis, and connectome-based predictive modeling with repeated 10-fold cross-validation was used to predict post-training Go-choice bias. Participants selected Go items above chance after training (59.3%). Whole-brain Go—No-Go connectivity showed modest cross-validated concurrent prediction of post-training Go-choice bias (combined network: mean ρ = 0.408, RMSE = 0.169, *p* < 0.001; positive network: ρ = 0.325, RMSE = 0.174, *p* < 0.001; negative network: ρ = 0.394, RMSE = 0.169, *p* < 0.001). Positive predictive edges prominently linked medial frontal and frontoparietal systems with the motor network and connected regions within the default mode network; medial frontal–salience connections were prominent in the distributed negative network. These findings identify candidate predictive connectivity patterns associated with individual variability in post-training Go-choice bias that require independent validation.

## 1. Introduction

Modern environments are saturated with palatable, energy-dense foods—particularly those high in sugar and fat (Hill et al., 2003)—and resisting them is a daily challenge for the many people who strive to eat more healthily (Birch & Fisher, 1998). A pivotal determinant of success is inhibitory control, the executive function that suppresses impulsive or prepotent responses in the service of personal standards and long-term goals (Diamond, 2013). At the neural level, inhibitory control depends on coordinated activity among the dorsolateral prefrontal cortex (DLPFC), right inferior frontal gyrus (rIFG), pre-supplementary motor area (pre-SMA), and dorsal anterior cingulate cortex (dACC), together with striatal structures and the subthalamic nucleus (STN) under dopaminergic modulation—circuitry that supports conflict monitoring, action gating, and outright stopping (Cools, 2008; Ridderinkhof et al., 2004; Criaud & Boulinguez, 2013). Consistent with this frontostriatal gating account, working-memory gating paradigms (update vs. ignore) likewise recruit the DLPFC and related frontostriatal circuitry in diet- and obesity-relevant samples (Hartmann et al., 2023; Herzog et al., 2024). In dietary self-control, these control systems interface with valuation and reward regions (e.g., ventral striatum, orbitofrontal cortex, ventromedial prefrontal cortex [vmPFC]) (Hare et al., 2009; Bartra et al., 2013) and with default mode network (DMN) hubs (e.g., vmPFC, posterior cingulate cortex) that support self-referential processing and the construction of subjective value (Andrews-Hanna et al., 2014).

Lower inhibitory control is associated with more uncontrolled eating (Dohle et al., 2018; Nelson et al., 2020) and higher body mass index (BMI) (Yang et al., 2018). It is therefore unsurprising that domain-general inhibition training—using go/no-go or stop-signal tasks—has been widely deployed to strengthen inhibitory control and, in turn, to modify eating behavior and body weight (e.g., Aulbach et al., 2020; Forman et al., 2019; Houben & Jansen, 2011; Lawrence et al., 2015b; Mann & Ward, 2024). Such protocols typically pair go/stop demands with largely arbitrary cues in the hope of up-regulating a general-purpose capacity. Yet evidence that this approach produces far transfer to eating behavior is weak, plausibly because of task-specific learning, task impurity, and outcome–measure mismatch (Beauchamp et al., 2016; Lawrence et al., 2015b; Sala et al., 2018).

Food go/no-go training takes a different approach: it pairs food images with response-contingent cues—often disproportionately assigning No-Go signals to high-calorie items—to forge item-contingent inhibitory associations. This was the paradigm adopted in the present study: a colored rectangular frame around each food image signaled the required response (green = press for Go; gray = withhold for No-Go), with Go and No-Go trials equiprobable (1:1). To avoid confusion, we use the term ‘food go/no-go training’ to mean speeded execution versus withholding of responses to food images (button press vs. no press); it does not involve approach–avoidance movements (e.g., push/pull; see Mehl et al., 2018, 2019).

Relative to domain-general inhibition training, food go/no-go training has been shown to alter food evaluations and wanting (e.g., Z. Chen et al., 2016, 2018a, 2018b; Houben, 2023; Yang et al., 2022, 2026), preferences (e.g., Z. Chen et al., 2019; Wu et al., 2023; Xia et al., 2024), intake (e.g., Lawrence et al., 2022; Yang et al., 2019), and, in some cases, body weight or adiposity (e.g., Lawrence et al., 2015a; Stice et al., 2017; Veling et al., 2014; but see Adams et al., 2021; Carbine & Larson, 2019; Yang et al., 2023). Across preregistered experiments, for example, training has repeatedly increased the probability of choosing trained Go items over matched No-Go items and reduced evaluations of No-Go items in post-training choice tasks (Z. Chen et al., 2019, 2021; Z. Chen & Veling, 2022; Wu et al., 2023; Xia et al., 2024).

Two mechanistic accounts have been proposed to explain these effects. The value-updating account holds that training rewrites valuations: No-Go foods are devalued, whereas Go foods accrue value (Veling et al., 2022). The stimulus–response account instead holds that training forges associations linking Go foods to action and No-Go foods to stopping, producing a “learned reflex” that can steer subsequent choices and preferences (Johannes et al., 2021; Veling et al., 2017; Verbruggen et al., 2014; Verbruggen & Logan, 2008).

Neuroimaging has begun to illuminate the mechanisms behind these effects. Recent fMRI studies have examined the neural correlates of training-related changes in food evaluation (Nakamura et al., 2023; Yang et al., 2023, 2026). Yang et al. (2023) found that lower explicit evaluations of No-Go foods after training were accompanied by reduced BOLD responses in the mid-insula and middle frontal gyrus when those items were viewed. Nakamura et al. (2023) observed higher evaluations for Go foods, lower desire-to-eat ratings for No-Go foods, increased mid-ventral insula and pons responses to Go items, and training-related connectivity changes. Most recently, Yang et al. (2026) showed that training selectively reduced wanting for No-Go foods, an effect tracked by decreased responses in the middle frontal, middle occipital, and inferior occipital gyri and by altered effective connectivity among these regions. Two further fMRI studies examined training-related differences during food choice itself (Wu et al., 2023; Xia et al., 2024): stronger Go-choice tendencies were associated with weaker recruitment of cognitive-control (e.g., DLPFC) and salience (e.g., dACC) networks for Go > No-Go selections—consistent with the reduced control and monitoring demands predicted by a stimulus–response account, although valuation contributions cannot be excluded. Together, these studies identify candidate nodes and underscore the importance of task context (evaluation vs. choice). However, they have largely relied on regional amplitudes and ROI-restricted connectivity, leaving open whether distributed coupling across large-scale brain networks during choice accounts for individual differences in post-training Go-choice bias.

To our knowledge, no prior study has directly tested whether task-modulated whole-brain connectivity predicts individual differences in post-training Go-choice bias. We therefore asked whether distributed interregional coordination during the decision window provides cross-validated concurrent prediction of post-training Go-choice bias beyond regional activation amplitudes. The two mechanistic accounts make distinguishable network-level predictions: value updating should recruit valuation/reward and salience systems, whereas a stimulus–response mechanism should implicate default-mode and memory systems that reinstate internalized cue–action associations to guide choice; both accounts require control networks. The concurrent design can identify which systems carry predictive information but cannot distinguish between the value-updating and stimulus–response accounts. Related work on nonreinforced training has documented network-level reconfiguration over time, providing a systems-level rationale for connectivity analyses (Itzkovitch et al., 2025). Operationally, we estimated ROI-to-ROI generalized psychophysiological interactions (gPPI) during a food-choice task, applied whole-brain connectome-based predictive modeling (CPM) with cross-validation and permutation testing to predict post-training Go-choice bias, and aggregated CPM-selected edges onto canonical brain systems to evaluate the involvement of valuation/reward, default-mode, and control networks in the predictive pattern.

## 2. Materials and Methods

### 2.1. Participants

A total of 174 female undergraduate and graduate students were enrolled through on-campus advertisements. This study was not preregistered, and no a priori power analysis was conducted; we recruited as many participants as feasible within budget and timeline constraints. Only women were included to reduce sample heterogeneity related to sex differences in eating behavior (B. J. Rolls et al., 1991) and metabolism (Blaak, 2001). Recruitment feasibility was an additional consideration: the volunteer pool at Southwest University was predominantly female, and recruitment of sufficient male participants had been difficult in our previous MRI studies. Participants were excluded if they had existing mental disorders, eating disorders, or head injuries. All participants were instructed to abstain from eating for 3 h before arriving at the laboratory (water was allowed).

Two participants were excluded from the analysis: one demonstrated extremely low accuracy (below 80%) in the food go/no-go training task, and one dropped out of the experiment. The 80% training-accuracy cutoff was established before examining the present data, following the laboratory protocol used in our previous food go/no-go training study (Wu et al., 2023). This criterion was not preregistered. The imaging data of two participants were unavailable due to scan errors, and the imaging data of eight participants were excluded from the analysis because of motion (displacement > 3 mm in any plane or rotation > 3° in any direction). As a result, our analyses included 172 participants for the behavioral data and 162 participants for the imaging data. Among the 174 participants, a subset completed a food-picture viewing task both before and after the training; those data are not pertinent to the current study. The research received approval from the local Ethics Committee (H22063) and was carried out in accordance with the Declaration of Helsinki.

### 2.2. Procedure

**Figure 1:**
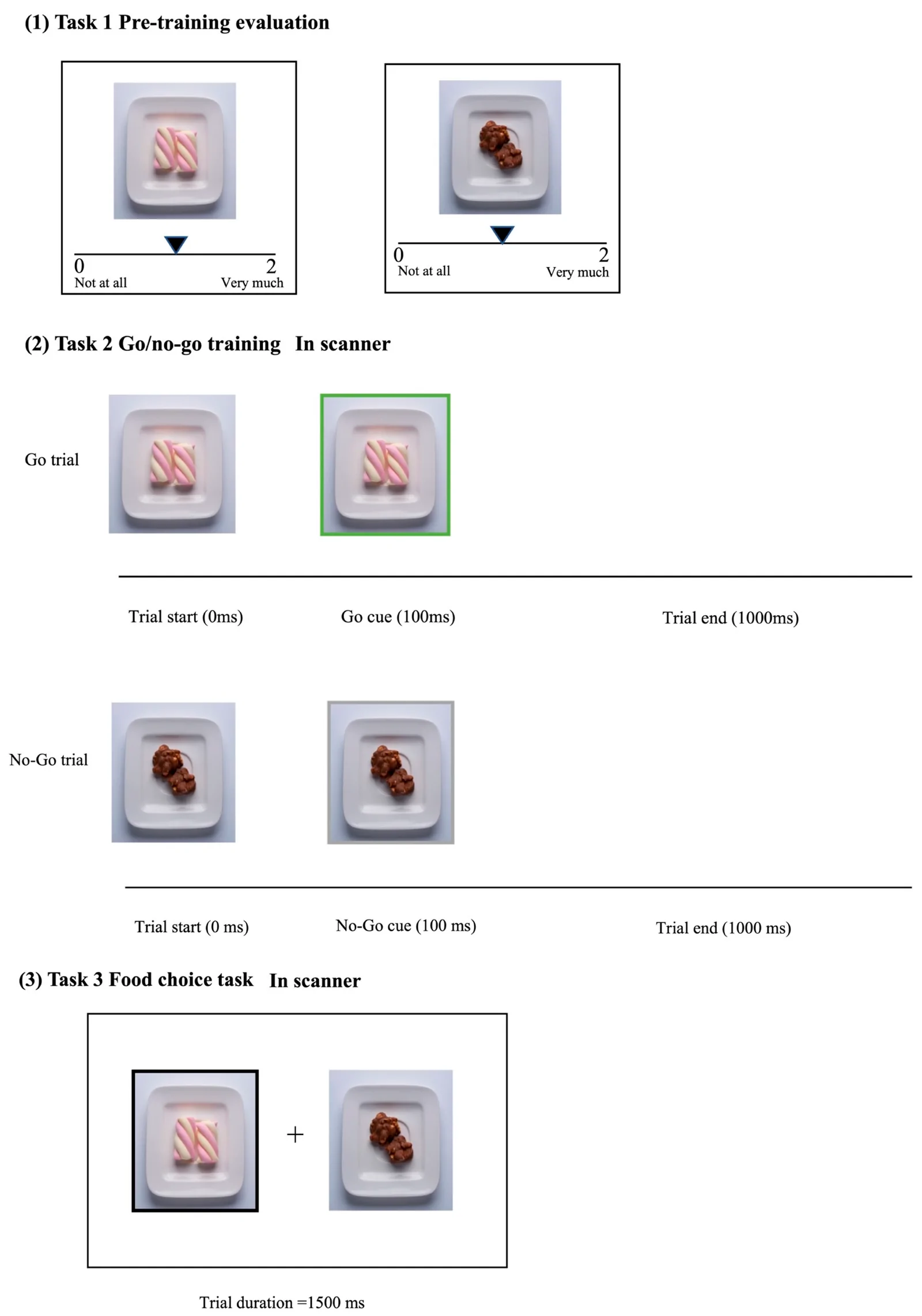
The sequence of main experimental tasks.

Figure 1 illustrates the study procedures. Upon arrival, participants provided informed consent and completed demographic questionnaires. They then practiced the food go/no-go training task and the binary food-choice task before proceeding to the formal experimental tasks.

### 2.3. Measurements

#### 2.3.1. fMRI Data Acquisition

Structural and functional images were acquired on a 3T Siemens Prisma MRI scanner (Siemens Healthcare GmbH, Erlangen, Germany) using a 64-channel brain coil at Southwest University. High-resolution structural images were acquired using a magnetization-prepared rapid acquisition gradient-echo (MPRAGE) sequence: repetition time (TR) = 2530 ms, echo time (TE) = 2.98 ms, field of view (FOV) = 224 × 256 mm^2^, resolution matrix = 448 × 512, flip angle = 7°, slices = 192, thickness = 1.0 mm, inversion time = 1100 ms, voxel size = 0.5 × 0.5 × 1 mm^3^. fMRI data were obtained using a single-shot echo-planar imaging (EPI) sequence (TR = 2000 ms; TE = 30 ms; flip angle = 90°; FOV = 224 × 224 mm^2^; matrix size = 112 × 112; voxel size = 2.0 × 2.0 × 2.0 mm^3^; slices = 62; thickness = 2.0 mm).

#### 2.3.2. Pre-Training Food Evaluation

During the evaluation task, a sequence of 40 color images of candies was presented on the screen in random order. These pictures were selected from a prior study (Z. Chen et al., 2019). For each picture, participants rated their desire to eat the depicted candy at that moment using a continuous 0–2 slider scale anchored at 0 = not at all and 2 = very much (Z. Chen et al., 2019). The food image remained on the screen until participants pressed a key (“Continue”) to confirm their rating and proceed to the subsequent trial. This behavioral rating task was administered outside the MRI scanner.

#### 2.3.3. Item Selection

Following the pre-training evaluations, each participant’s 40 candy pictures were rank-ordered from 1 = most preferred to 40 = least preferred. Consistent with established protocols (Z. Chen et al., 2019; Schonberg et al., 2014), we did not use the most extreme items; instead, 12 pictures (ranks 3–14) were selected. These 12 items were split into two subgroups with identical mean rank to value-match the Go and No-Go sets. Within each participant, six items (ranks 3, 6, 7, 10, 11, 14) were paired with the Go cue (Go items), and the remaining six (ranks 4, 5, 8, 9, 12, 13) were paired with the No-Go cue (No-Go items). This value matching was intended to minimize systematic baseline value differences in the measured ratings. It did not establish that pre-training Go-choice probability was exactly 50% or eliminate possible baseline differences in subsequent binary choice.

#### 2.3.4. Food Go/No-Go Training

The parameters of the training task closely aligned with those of previously published studies (Z. Chen et al., 2019; Wu et al., 2023; Yang et al., 2023; Xia et al., 2024). The set of 12 evaluated food images was used in the training. On each trial, a food image appeared centrally on a computer screen for up to 1 s, followed by a 1–4 s interstimulus interval. A rectangular frame, either green or gray, appeared around the picture 100 ms after picture onset and remained visible for the duration of the picture display. Participants were instructed to press a button on an MRI-compatible response box promptly and accurately if the rectangular frame was green (Go). If the rectangular frame was gray (No-Go), they were instructed to withhold their response. The training session comprised 12 runs, each consisting of 12 trials (6 Go and 6 No-Go trials per run), resulting in a total of 144 trials. The session took approximately 12 min to complete. Participants were permitted a break after completing half of the training session. Training was performed inside the MRI scanner.

#### 2.3.5. Binary Food Choice Task

This task assessed post-training Go-choice bias. No pre-training binary-choice task was administered. The 12 evaluated food images were employed. In each trial, one Go food and one No-Go food were presented side by side, and participants had to choose the food they desired to eat within 1.5 s by pressing the corresponding button on an MRI-compatible response box. After each selection, a black rectangle appeared around the chosen food for 500 ms as confirmation. The task comprised two runs, each with 36 trials. Between trials, a fixation cross was presented for an average of 2.5 s (range: 1–4 s). The total task duration was approximately seven minutes. Participants were given a break after completing half of the trials. The binary choice task was performed inside the MRI scanner. Choices were not used for consumption after the scan.

### 2.4. Statistical Analyses

#### 2.4.1. Behavioral Data Analysis

Behavioral data were analyzed in R 4.2.1 (R Core Team, 2013) using glmer from lme4 (Bates et al., 2015; https://CRAN.R-project.org/package=lme4, accessed on 29 September 2026). Pre-training ratings (Section 2.3.3) were used to construct value-matched Go and No-Go item sets for each participant; we defined post-training Go-choice bias under matched baseline value as the deviation of Go choices from the 50% reference in the binary probe. The dependent variable was the trial-level binary choice in the post-training probe (Go = 1, No-Go = 0). Because each probe trial paired value-matched Go and No-Go items, no trial-level predictors of baseline value were entered in the primary model. The model was fit at the trial level with participant as the grouping factor and age, BMI, and hunger as participant-level covariates (choice~age + BMI + hunger + (1|SubjectID)); the Go-choice bias was interpreted relative to chance (50%). For CPM, the target was each participant’s observed post-training Go-choice proportion across all probe trials. Because pre-training ratings and post-training binary choices were different measures, this outcome was not a directly observed pre-to-post change score; the 50% reference was not an observed pre-training choice probability.

#### 2.4.2. fMRI Data Processing and Task-Based Connectivity (gPPI)

Preprocessing of fMRI data was conducted using the DPABI toolbox (Yan et al., 2016; https://rfmri.org/DPABI, accessed on 29 September 2026) in MATLAB (R2021a, MathWorks, Natick, MA, USA). Procedures included slice-timing correction and head-motion correction, coregistration, segmentation, normalization to Montreal Neurological Institute space with voxel sizes of 3 × 3 × 3 mm^3^, and spatial smoothing with a Gaussian kernel of 8 mm full width at half maximum.

First-level analysis. First-level analyses were performed using SPM12. At the subject level, we modeled two conditions in the binary food-choice task—choosing Go foods and choosing No-Go foods—as decision-locked stick functions time-locked to the button press and convolved with the canonical HRF, together with six motion regressors and a session constant. Because a Go item and a No-Go item were presented simultaneously on each trial, generic viewing/anticipation was shared across items and could not be uniquely attributed to either category; therefore, separate viewing/anticipation regressors were not included.

ROI-to-ROI gPPI. The functional/structural volumes and condition information from the SPM first-level design files were imported into the CONN toolbox (version 22; Whitfield-Gabrieli & Nieto-Castanon, 2012) for ROI-to-ROI gPPI analysis. Consistent with previous studies (e.g., Ovando-Tellez et al., 2022), we used the Shen atlas with 268 nodes. We selected “gPPI analysis of selected task factors” as the analysis type and specified the bivariate correlation option, which applies Fisher transformation to normalized PPI coefficients. For each ROI pair, CONN estimated condition-specific connectivity for Go-choice and No-Go-choice based on the physiological time series, the psychological regressors, and their interaction (PPI) terms, with standard denoising and motion confounds. Each condition matrix was symmetrized by averaging the two directions of each ROI pair. We then computed a Go—No-Go difference matrix (268 × 268) for each participant, which served as the task-modulated connectivity input for CPM. The gPPI GLM was fit to the full acquired choice-task time series (232 volumes; TR = 2 s), so all volumes contributed to estimation; the analysis did not rely on correlating short time-series snippets. Connectivity was estimated using all valid Go-choice and No-Go-choice events; the numbers of events were not matched between conditions.

#### 2.4.3. Connectome-Based Predictive Modeling

CPM was implemented using published MATLAB scripts (Shen et al., 2017) with feature screening and model fitting within each training fold. For each participant, the off-diagonal values of the gPPI-derived Go−No-Go matrix were standardized across edges by subtracting their mean and dividing by their sample standard deviation. This normalization used only that participant’s connectivity values. The upper triangle, excluding the diagonal, was then extracted to yield 35,778 edge-wise features. Within each training fold (i.e., without access to the held-out data), we computed partial Spearman correlations between every edge and the post-training Go-choice proportion (Section 2.4.1), controlling for age, mean framewise displacement, BMI, and hunger; Spearman rank was used because post-training Go-choice proportions were non-normally distributed (Kolmogorov-Smirnov *p* < 0.05). Six fixed edge-selection thresholds (*p* < 0.05, 0.01, 0.005, 0.001, 0.0005, and 0.0001) were evaluated in separate repeated cross-validation runs following Shen et al. (2017). This threshold comparison used mean combined-network Spearman ρ and was not nested within an additional outer cross-validation loop. The *p* < 0.05 threshold was used for the main analysis; Supplementary Figure S1 summarizes performance across the fixed thresholds. CPM aggregates information across selected edges (see also Suo et al., 2022). Surviving edges were partitioned into positive and negative networks according to the sign of their partial association with behavior. Network strength was the sum of selected edge weights, and combined-network strength was the positive-network sum minus the negative-network sum. Separate simple linear models with an intercept predicted post-training Go-choice proportions from positive, negative, or combined network strength. Positive and negative network labels refer to edge–behavior associations, not the sign of connectivity or the choice condition. Because the connectivity features and behavioral outcome were derived from the same task, this analysis provides cross-validated concurrent prediction across participants rather than prediction of a later training response.

**Figure 2:**
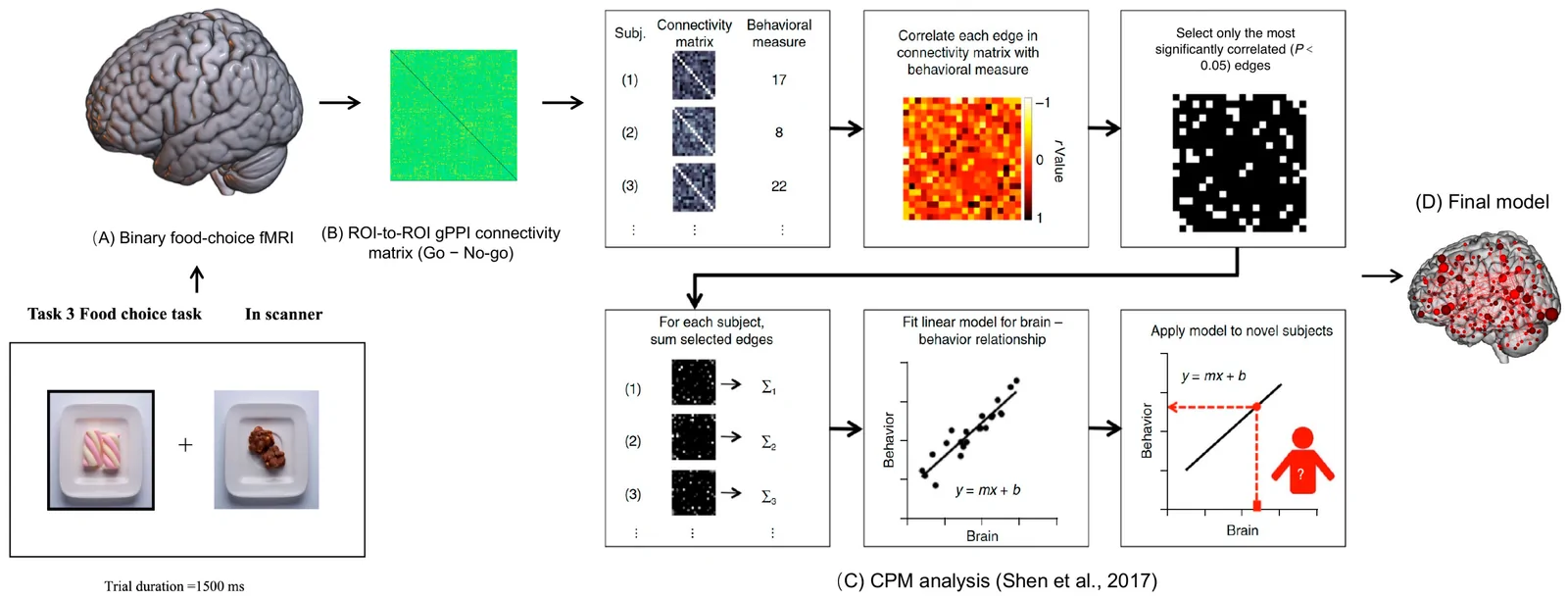
Pipeline for predicting post-training Go-choice bias from task-based connectivity. (**A**) Binary food-choice fMRI. (**B**) ROI-to-ROI gPPI using the Shen-268 atlas, followed by computation of a difference matrix between the Go-choice and No-Go-choice conditions. (**C**) CPM feature selection and positive, negative, and combined network strength (Shen et al., 2017). (**D**) Prediction with 10-fold cross-validation repeated 100 times and permutation testing.

To mitigate overfitting and obtain low-variance generalization estimates, we used repeated 10-fold cross-validation (CV) with fully nested feature screening. In our sample (*N* = 162), leave-one-out cross-validation (LOOCV) would yield training sets that overlap by approximately 99.4% (160/161) and single-participant test folds; by contrast, 10-fold CV uses approximately 10% test folds (16–17 participants), producing more stable fold-level performance estimates. We repeated the 10-fold CV procedure 100 times with different random partitions and averaged held-out Spearman ρ and root mean squared error (RMSE) across repetitions. Within each repetition, performance was calculated across all 162 held-out predictions. Means, medians, and 25th–75th percentile intervals summarize performance across the 100 repetitions. Covariate control, feature selection, and model fitting were confined strictly to training folds; selected edges and fitted parameters were then applied to the held-out fold. For interpretability (not for model training), we quantified feature stability across all 1000 training folds (100 repetitions × 10 folds), labeling edges selected in at least 900 folds (≥90%) as stable. The ≥90% cutoff was an interpretive reporting threshold, rather than a preregistered criterion. It defined a consistent set of frequently selected connections for anatomical description; predictive models retained their training-fold-specific feature sets. This stable set was used for the anatomical summaries reported in Section 3.4. The complete analysis pipeline is illustrated in Figure 2.

Two sensitivity analyses examined the influence of pronounced imbalance between Go- and No-Go-choice trial counts. The first required at least two valid choices in each condition, excluding four participants and retaining 158 participants. The second required at least five valid choices in each condition, excluding eight participants in total and retaining 154 participants. Both analyses retained the connectivity inputs, covariates, and fixed edge-selection threshold (*p* < 0.05) used in the main analysis. Feature selection and model fitting were repeated within each training fold of 10-fold cross-validation, repeated 100 times.

A BMI sensitivity analysis excluded participants with BMI ≥ 25 kg/m^2^. Ten participants met this criterion in the behavioral sample; one had already been excluded from the imaging sample, leaving 153 participants for CPM. This exclusion was applied separately from the choice-condition imbalance exclusions. The analysis retained the same covariates and fixed edge-selection threshold (*p* < 0.05), with feature screening and model fitting repeated within each training fold across 100 repetitions of 10-fold cross-validation.

For the main analysis and the three exclusion-based sensitivity analyses, significance was assessed using 1000 permutations of the post-training Go-choice proportion across participants in the corresponding sample. Each permutation repeated the full 100 repetitions of 10-fold cross-validation using the same partitions as the observed analysis. The edge-selection threshold was fixed at *p* < 0.05, and the covariates were the same as in the observed analysis. Feature selection and model fitting were repeated within each training fold. The test statistic was the mean Spearman ρ across the 100 repetitions. Upper-tail permutation *p* values were calculated as (b + 1)/1001, where b is the number of permuted statistics greater than or equal to the observed statistic, with significance set at *p* < 0.05. Permutation *p* values were not adjusted for comparisons across the three networks.

Two further sensitivity analyses used the full imaging sample (*N* = 162). First, Go- and No-Go-choice event counts were matched within participant by randomly subsampling the larger condition, and gPPI was re-estimated for five draws while modeling unselected events separately. Second, CPM used Go-condition connectivity from the original full-event gPPI model without subtracting No-Go connectivity. Both analyses retained the original behavioral outcome and CPM settings; inference used 1000 permutations, each repeating 100 repetitions of 10-fold cross-validation. For the matched analysis, the test statistic was averaged across all five draws. Detailed methods are provided in Supplementary Sections S6 and S7.

## 3. Results

### 3.1. Demographic Information

The sample had a mean age of 20.53 years (SD = 1.49; range = 18–26 years) and a mean BMI of 20.70 (SD = 2.74; range = 15.63–31.14); ten participants were classified as overweight.

### 3.2. Behavioral Results

#### 3.2.1. Performance During Food Go/No-Go Training

Participants made very few errors during training (mean accuracy = 98.90%, SD = 1.83%), and the mean Go response time was 382.40 ms (SD = 45.96 ms).

#### 3.2.2. Pre-Training Evaluation and Value Matching

Before training, participants rated all 40 food images, and we constructed Go and No-Go sets with equal mean ranks for each participant (within-participant matching). A paired-samples *t* test showed no significant difference in pre-training ratings between the Go (M = 1.32, SD = 0.40) and No-Go (M = 1.32, SD = 0.40) sets, t(171) = 0.77, *p* = 0.445.

#### 3.2.3. Probe in Food Choice Task

**Figure 3:**
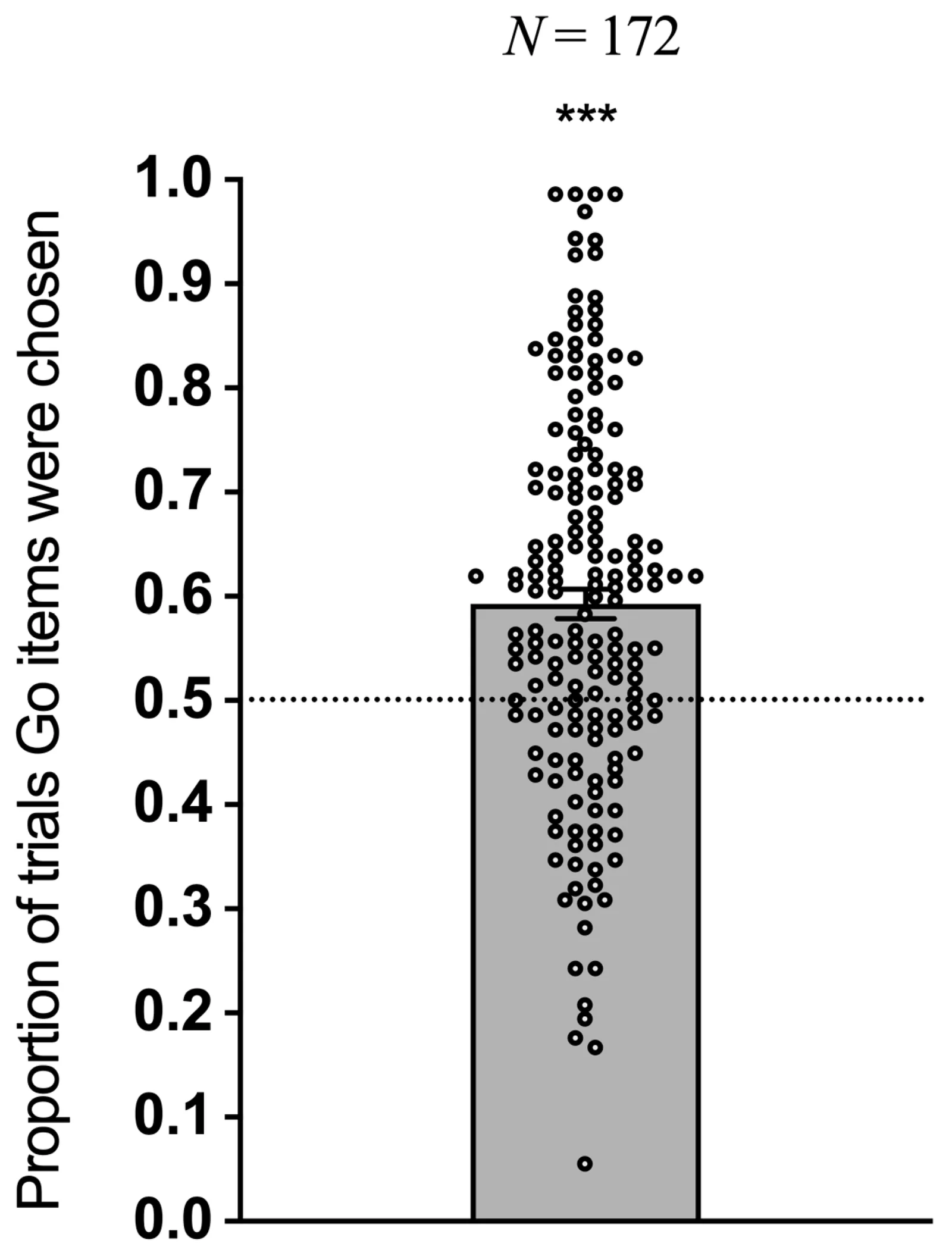
Behavioral results for Go-food choices during the binary food-choice task. The mean proportion of trials in which participants chose Go over No-Go foods is presented. The dashed line indicates the chance level of 50%; asterisks above the dashed line indicate a proportion higher than chance; error bars represent the standard error of the mean. *** *p* < 0.001.

Participants chose Go items on 59.30% of post-training trials, above the 50% reference level (OR = 1.58, 95% CI [1.37, 1.82], *p* < 0.001; Figure 3). This post-training Go-choice bias is consistent with a training-related preference effect under matched baseline ratings.

### 3.3. Predictive Efficacy of the Model

**Figure 4:**
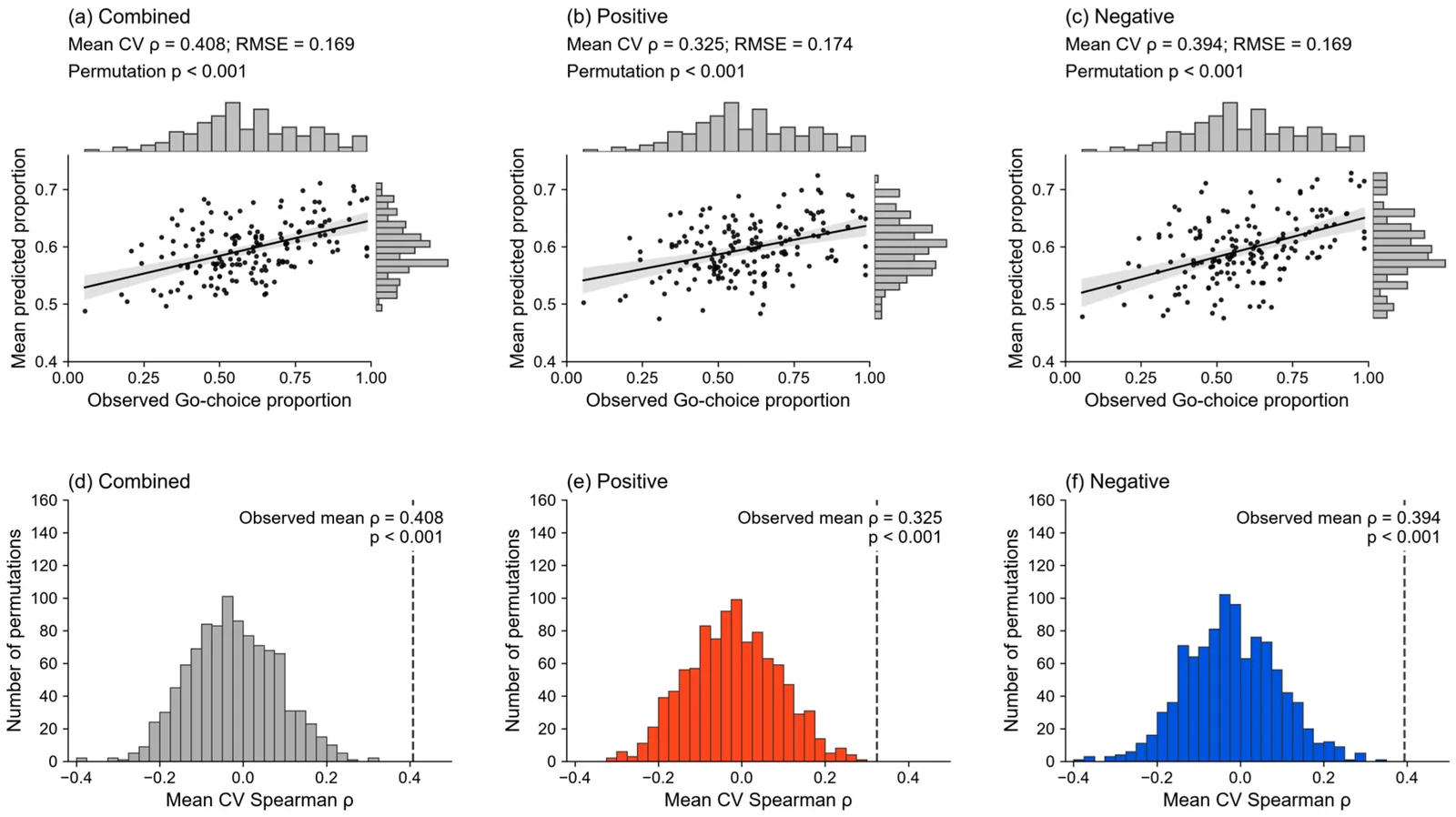
Predictive performance of the model. Panels (**a**–**c**) show observed post-training Go-choice proportions and held-out predictions averaged across 100 repetitions for the combined, positive, and negative networks, respectively. Lines show descriptive linear fits with 95% confidence bands. Annotations report the mean within-repetition Spearman ρ and RMSE and the upper-tail permutation *p* value. Panels (**d**–**f**) show the null distributions of mean Spearman ρ obtained from 1000 outcome permutations for the corresponding networks. Each permutation repeated the full 100 repetitions of 10-fold cross-validation. Dashed lines indicate the observed mean Spearman ρ.

With the Go—No-Go connectivity matrix as input, whole-brain CPM showed modest cross-validated concurrent prediction of post-training Go-choice bias (combined network: mean ρ = 0.408, RMSE = 0.169, *p* < 0.001, Figure 4a,d; positive network: ρ = 0.325, RMSE = 0.174, *p* < 0.001, Figure 4b,e; negative network: ρ = 0.394, RMSE = 0.169, *p* < 0.001, Figure 4c,f).

For the combined network, median Spearman ρ was 0.406 (25th–75th percentiles: 0.390–0.427), and median RMSE was 0.1692 (0.1681–0.1701). The corresponding median correlations were 0.322 (0.302–0.354) for the positive network and 0.395 (0.374–0.412) for the negative network. Performance summaries for all three networks are provided in Supplementary Table S1.

After excluding participants with fewer than two valid choices in either condition (*N* = 158), mean Spearman correlations were 0.386, 0.348, and 0.343 for the combined, positive, and negative networks, respectively (permutation *p* < 0.001, *p* < 0.001, *p* = 0.002). When at least five choices in each condition were required (*N* = 154), mean Spearman correlations were 0.270, 0.280, and 0.195 for the combined, positive, and negative networks, respectively (permutation *p* = 0.005, *p* = 0.003, *p* = 0.023). Predictive associations remained positive and significant across all three networks in both sensitivity analyses (Supplementary Figure S2). These exclusions address extreme imbalance but do not equalize trial counts between conditions.

After excluding participants with BMI ≥ 25 kg/m^2^ (*N* = 153), mean Spearman correlations were 0.425, 0.311, and 0.430 for the combined, positive, and negative networks, respectively (all permutation *p* < 0.001). Mean RMSE values were 0.173, 0.179, and 0.170, respectively. Predictive associations remained positive and significant across all three networks (Supplementary Figure S3).

After within-participant trial-count matching, aggregate mean Spearman correlations across five draws were 0.001, 0.056, and −0.039 for the combined, positive, and negative networks (permutation *p* = 0.361, 0.170, and 0.579). Using Go-condition connectivity alone, the corresponding correlations were 0.314, 0.170, and 0.365 (*p* < 0.001, *p* = 0.043, and *p* < 0.001). Full results are reported in Supplementary Sections S6 and S7 (Tables S2 and S3).

### 3.4. Post-Training Go-Choice Bias-Related Network Neuroanatomy

**Figure 5:**
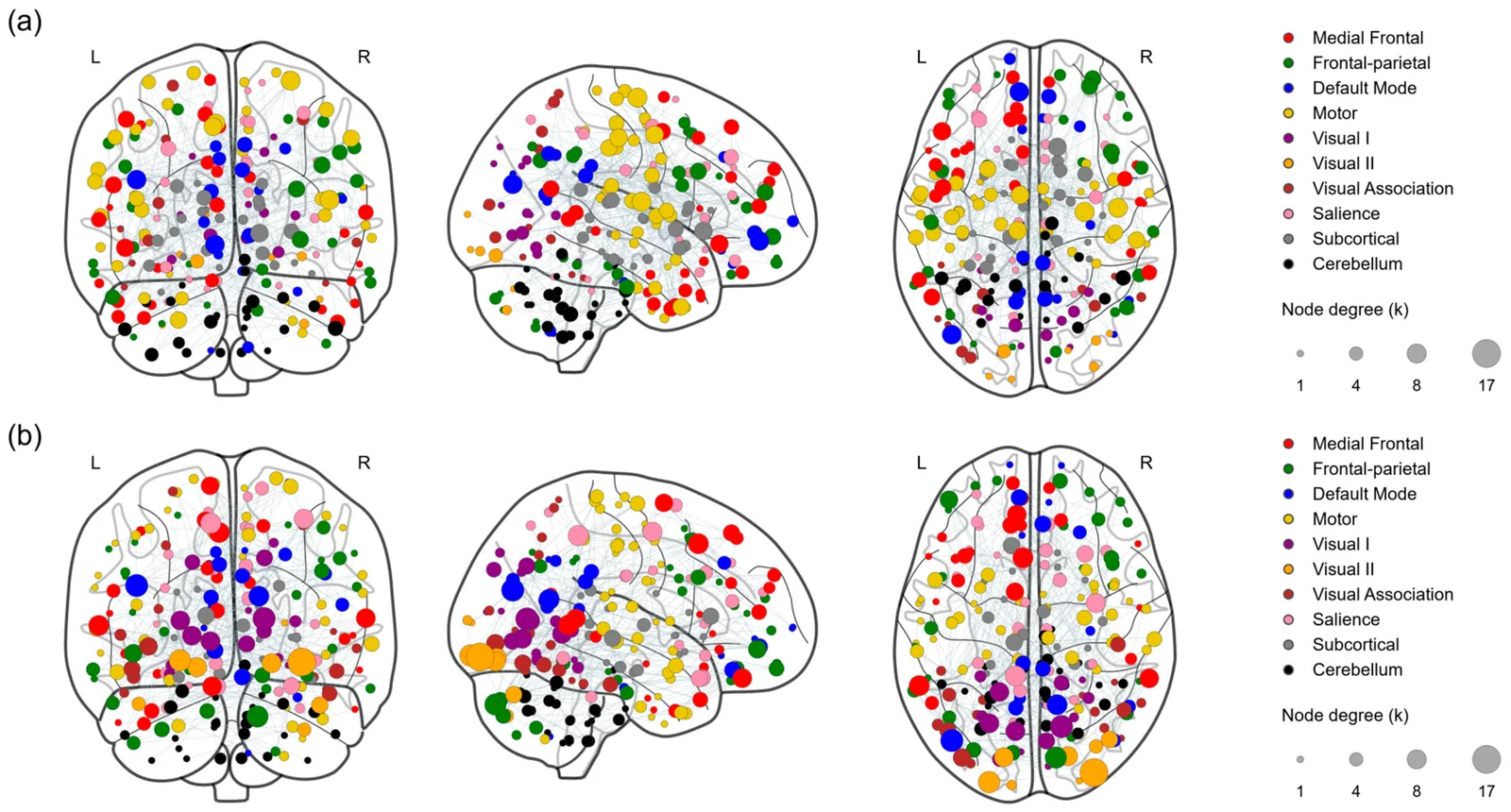
Positive (**a**) and negative (**b**) networks predicting individual differences in post-training Go-choice bias. Only stable edges, selected in at least 900 of the 1000 training folds, are shown. Node colors indicate canonical networks; node area is proportional to degree (k), with the same scale used in both panels. L = left; R = right.

The CPM-identified predictive network spanned connections within and between multiple brain regions. At the ≥90% selection-frequency threshold, the stable connectivity masks contained 1332 selected off-diagonal entries (616 positive and 716 negative; Figure 5), representing 1.86% of the possible off-diagonal entries in the Shen-268 connectivity matrix. Because the matrices were symmetric, these entries corresponded to 666 unique undirected edges: 308 positive and 358 negative. The anatomical summaries below use this stable set, whereas predictive fitting used the fold-specific feature sets.

Table 1 and Table 2 list the ten highest-degree nodes in the positive and negative networks, respectively. In the positive network (Figure 5a), the most connected nodes included the right precentral gyrus and insula in the motor network (both k = 8), the left medial orbitofrontal and middle occipital gyri in the default mode network (both k = 8), the right caudate in the subcortical network (k = 7), the left inferior frontal gyrus in the medial frontal network (k = 7), and two left middle cingulate parcels in the motor network (both k = 7). In the negative network (Figure 5b), the highest-degree node was the right inferior occipital gyrus in the visual II network (k = 17), followed by the right calcarine cortex in visual I and the left middle occipital gyrus in the default mode network (both k = 11). Other highly connected nodes included right cerebellar Crus II in the frontoparietal network and the left inferior occipital gyrus in visual II (both k = 10), as well as medial frontal and salience parcels (k = 9). Functional network assignments followed the ten-system Shen-268 parcellation distributed with BioImage Suite (Finn et al., 2015; Noble et al., 2017); anatomical names describe the locations of parcel centroids. SAL and SC denote the limbic and basal-ganglia subdivisions, respectively, in the atlas metadata.

**Figure 6:**
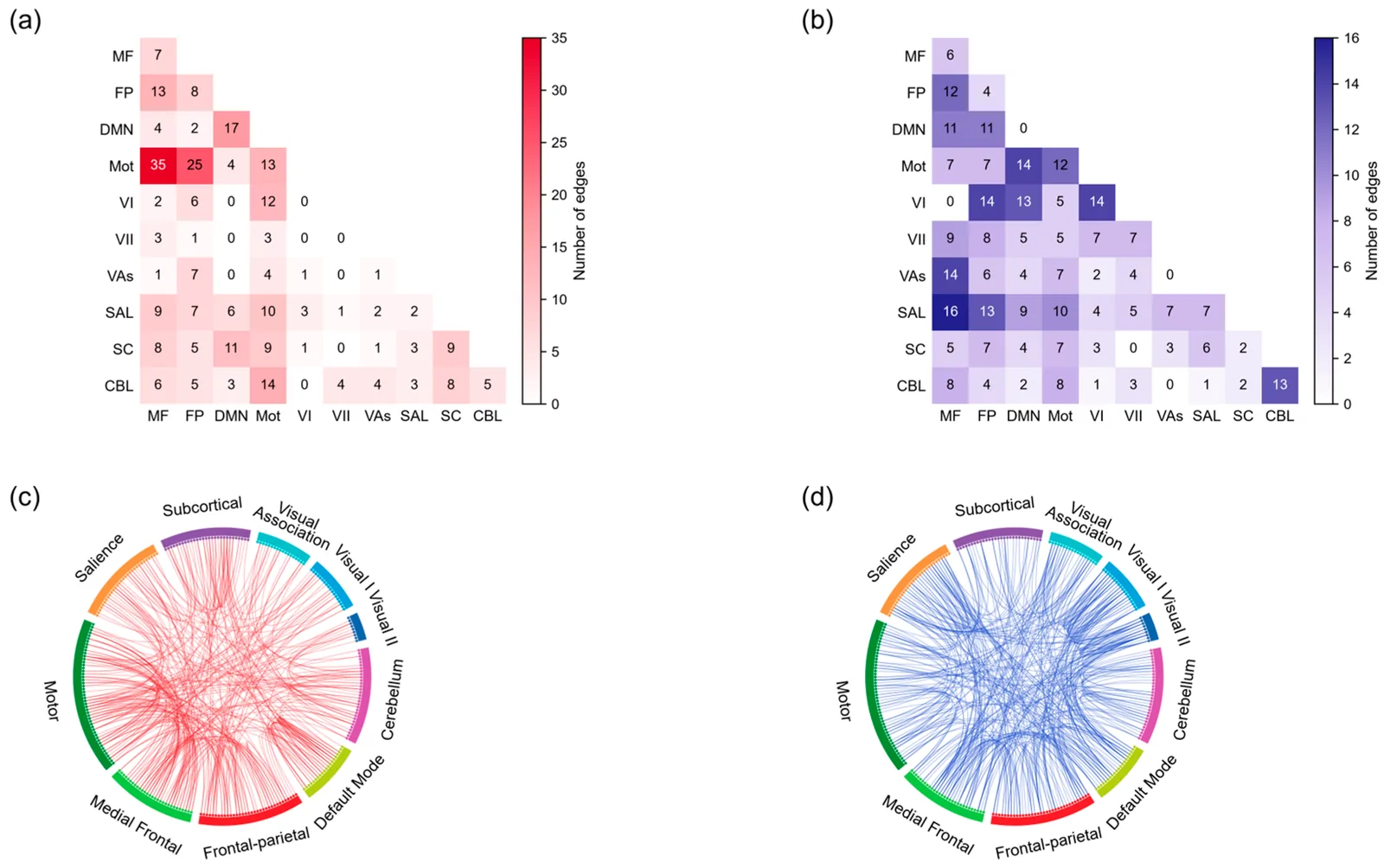
Within- and between-network connectivity for the positive (**a**) and negative (**b**) networks. Cells represent the total number of unique undirected edges connecting nodes within and between each canonical network, with darker colors indicating more edges; diagonal cells count connections between distinct nodes within the same network. Connections for the positive and negative networks are shown in (**c**) and (**d**), respectively. CBL = cerebellar network; SC = subcortical network; SAL = salience network; VAs = visual-association network; VII = visual network II; VI = visual network I; Mot = sensorimotor network; DMN = default mode network; FP = frontoparietal network; MF = medial frontal network.

Figure 6 summarizes the distribution of stable edges within and between canonical networks for the positive and negative networks. The positive network contained the largest numbers of connections between the medial frontal and motor networks (MF–Mot, 35 edges), between the frontoparietal and motor networks (FP–Mot, 25 edges), and within the default mode network (DMN–DMN, 17 edges; Figure 6a,c). The negative network contained the largest number of connections between the medial frontal and salience networks (MF–SAL, 16 edges), followed by DMN–Mot, FP–VI, MF–VAs, and within-VI connections (14 edges each; Figure 6b,d). Thus, MF–SAL connections formed a prominent component of a distributed negative network. These counts describe the distribution of stable edges and do not constitute tests of network enrichment.

## 4. Discussion

We applied connectome-based predictive modeling to a substantial task-based fMRI sample (*N* = 162) to examine cross-validated concurrent prediction of individual differences in post-training Go-choice bias. Task-evoked Go−No-Go connectivity showed modest prediction of post-training Go-choice bias, with mean Spearman correlations of 0.408, 0.325, and 0.394 for the combined, positive, and negative networks, respectively. Positive predictive edges prominently linked medial frontal and frontoparietal systems with the motor network and connected regions within the default mode network. Medial frontal–salience connections were prominent in the distributed negative network. These findings identify predictive associations compatible with possible roles for goal-directed action selection, internally guided cognition, and the motivational significance of food cues.

Behaviorally, participants preferred Go over No-Go foods after training, consistent with previous reports of training-related Go-choice preferences (Z. Chen et al., 2021; Z. Chen & Veling, 2022; Wu et al., 2023; Xia et al., 2024). Across this converging literature, repeated responding or response withholding to food stimuli without external reinforcement has been consistently associated with subsequent changes in preferences for those foods.

Several fMRI studies have probed the neural mechanisms of preference change induced by food go/no-go training (Wu et al., 2023; Xia et al., 2024) or by related nonreinforced paradigms, such as cue-approach training (Aridan et al., 2019; Botvinik-Nezer et al., 2020; Salomon et al., 2019; Schonberg et al., 2014; Schonberg & Katz, 2020) and approach–avoidance training (Mehl et al., 2019; Zahedi et al., 2023). In our own recent work, gPPI analyses showed that connectivity between the inferior parietal lobule and the pre-supplementary motor cortex, dACC, and middle frontal gyrus (Wu et al., 2023), or between the superior frontal gyrus and middle occipital gyrus (Xia et al., 2024), predicted preference modification, with stronger connectivity tracking larger change; likewise, training-induced reductions in food wanting were accompanied by decreased middle frontal and occipital responses and altered effective connectivity among these regions (Yang et al., 2026). In cue-approach training, vmPFC–superior temporal sulcus connectivity during training predicted preference change (Salomon et al., 2019), and in approach–avoidance training, increased activity in the posterior cingulate cortex—a core DMN hub—tracked preference shifts (Zahedi et al., 2023).

The present study offers several advantages over prior work. First, the behavioral sample was relatively large (*N* = 172), and the task-based fMRI CPM sample remained substantial (*N* = 162), increasing the reliability of the estimates for this literature. Second, to our knowledge, this is among the first studies to use a fully data-driven whole-brain approach to characterize individual differences in post-training Go-choice bias. Third, unlike earlier ROI-focused analyses, whole-brain CPM required no a priori network selection and yielded statistically significant but modest cross-validated prediction.

Consistent with prior CPM work (e.g., X. Chen et al., 2023), the network associated with post-training Go-choice bias exhibited distributed, cross-system connectivity. The positive network contained particularly many MF–Mot and FP–Mot connections. Medial frontal and frontoparietal systems contribute to maintaining goals and task rules, monitoring conflict, and selecting appropriate actions (Ridderinkhof et al., 2004; Menon & D’Esposito, 2022). Experimental evidence also links medial frontal–motor interactions to action selection under conflict (Mars et al., 2009). Their coordination with the motor network may therefore provide a route through which food-related goals and learned response tendencies influence the selection and execution of a choice. Under a stimulus–response account, repeated responding to Go foods could establish cue–action associations that are expressed during subsequent choice; MF–Mot and FP–Mot connectivity would be compatible with translating these associations into action. This interpretation does not require Go-choice bias to reflect stronger response inhibition during the probe: participants pressed a button for both Go and No-Go selections. The observed connections are predictive associations and do not establish that training altered action-control circuitry.

Within-DMN connections were another prominent component of the positive network. The DMN supports internally directed cognition, memory-based representations, and the construction of personally relevant meaning (Raichle, 2015; Smallwood et al., 2021). It can also participate in goal-directed cognition when internally represented information is relevant to the task (Spreng et al., 2010). In the present setting, within-DMN connectivity may be compatible with the reinstatement of learned food–response associations or internally represented goals that guide subsequent choice. One possibility is that repeated cue–response pairings allow task goals and action tendencies to become internalized, so that familiar food cues can elicit learned choice tendencies with less reliance on explicit deliberation. Together with the MF–Mot and FP–Mot pattern, this offers a possible systems-level account linking internally represented information to action selection. However, within-DMN connectivity alone does not demonstrate goal internalization, automaticity, or a training-induced change in these processes. Value updating, learned cue–action mappings, and action control remain possible interpretations that the concurrent design does not distinguish.

The negative network provides a complementary perspective. MF–SAL was its most frequent network pair, although predictive edges were distributed across several systems. Higher standardized Go−No-Go edge values in the negative network were associated with lower post-training Go-choice proportions. Salience-related processing can prioritize motivationally relevant cues and influence the allocation of control, but salience and reward value are not interchangeable constructs (Litt et al., 2011; E. T. Rolls, 2015). One possible interpretation is that No-Go foods retaining motivational or reward significance after training continue to compete with learned Go-linked action tendencies, thereby favoring No-Go choices in some individuals. MF–SAL involvement is compatible with control processes integrating such competing motivational information. This interpretation connects the negative network to the value-updating account, under which the extent of No-Go devaluation varies across individuals (Veling et al., 2022; Yang et al., 2022). The Go−No-Go contrast and the sign of an edge–behavior association do not identify No-Go-specific coupling or directly measure residual reward value. Testing this account would require item-level post-training value measures and analyses separating the two choice conditions.

Our CPM findings complement—but also diverge from—prior multivariate approaches to the neuroimaging of eating behavior. Multivariate machine-learning studies have mapped value- and control-related brain signals that shape eating: from fMRI activation patterns, Cosme et al. (2020) derived a “regulation signature” that generalized to uninstructed food choice and tracked lower craving and reduced subjective value of unhealthy foods; in the orbitofrontal cortex (OFC), Xue et al. (2022) decoded health and taste evidence from distributed patterns, with health evidence more tightly coupled to choice magnitude in anorexia nervosa; and using electroencephalography (EEG), Schubert et al. (2021) showed that health and taste attributes become decodable within the first second of cue processing, earlier than the decision outcome itself. Complementing these trial-level decoders, resting-state CPM has linked intrinsic whole-brain connectivity to eating-disorder symptom dimensions (X. Chen et al., 2023). In contrast, we trained a choice-locked, task-evoked CPM that predicts subject-level post-training Go-choice bias from whole-brain connectivity during the decision window—targeting transient network coordination at the moment of choice rather than static trait architecture or voxel-wise attribute codes, and thereby describing network-level associations with concurrent post-training choice behavior.

The practical implication is a set of candidate connectivity patterns for further study of individual differences in food choice after training. A next step would be to test, in an independent sample, whether connectivity measured before training predicts subsequent changes in the same choice measure assessed before and after training. Comparing active training with a control condition and measuring delayed choices and actual food intake would help establish whether any prediction is specific to training and relevant beyond the scanner. Only after such validation could these measures be evaluated for identifying individuals who may benefit from food go/no-go training or for adapting training protocols. The current model does not support individual treatment decisions.

Several limitations warrant caution. First, the predictive associations were modest (ρ ≈ 0.32–0.41); accordingly, the CPM-derived patterns should be considered candidate predictive connectivity patterns requiring independent validation rather than clinically actionable biomarkers. In addition, performance was compared across fixed edge-selection thresholds without an additional nested validation loop; the threshold comparison does not provide an independent estimate of the performance of a threshold-selection procedure. Second, models were evaluated with internal cross-validation only, without an independent replication sample, which limits inferences about generalizability; future work should test out-of-sample performance in preregistered, adequately powered studies. Connectivity and Go-choice proportion were measured during the same post-training task; the findings therefore concern concurrent prediction across participants rather than prediction of a later training response. Because the Go-choice and No-Go-choice conditions were defined by participants’ responses, differences in trial numbers may affect the precision of the connectivity estimates. Predictive associations remained positive and significant across all three networks after excluding participants with extreme choice-condition imbalance. Prediction was not significant after within-participant trial-count matching, which reduced the events assigned to the target conditions by 33.2%. This procedure may reduce estimation precision, but the contribution of information loss cannot be separated from the influence of trial-count imbalance. Go-only connectivity retained significant concurrent prediction while preserving all Go events. Together, these analyses do not establish independence from trial-count effects (Supplementary Sections S6 and S7). Third, we assessed immediate post-training choice effects and did not measure durability. Prior behavioral work suggests preference changes can persist for ~1 week (e.g., Z. Chen et al., 2019), but we did not collect delayed probes; longitudinal follow-ups (e.g., at 1 week and beyond) are needed to evaluate durability and whether CPM-identified connectivity predicts stability. Fourth, because participants were exclusively women (predominantly healthy-weight) and menstrual-cycle phase was not tracked, we cannot determine whether sex, adiposity, or cycle-related hormonal variation moderate either the CPM signatures or the impact of training. This limits generalizability to clinical and public-health contexts in which food-specific inhibitory-control training is intended to promote healthier choices. Notably, feasibility trials in binge-type eating disorders and studies in overweight samples suggest such training is acceptable and can shift food valuation and/or eating-related symptoms (e.g., Chami et al., 2022; Keeler et al., 2022; Stice et al., 2022; Yang et al., 2023). External validation across sex and BMI—and designs that standardize/model cycle phase—are needed. The predictive associations remained positive and significant after excluding participants with BMI ≥ 25 kg/m^2^, although residual BMI-related confounding cannot be excluded. Similarly, the absence of significant BMI prediction in an exploratory analysis (Supplementary Section S2) does not rule out a contribution of BMI to the neural–behavioral association. Fifth, we paired Go and No-Go items on pre-training value, a design that does not mirror real-world settings in which healthier options are often initially less liked. Pre-training desirability ratings and post-training binary choices were different measures, so the outcome did not directly quantify within-person preference change. Building on work showing that training can promote healthier choices under value imbalance (e.g., Z. Chen et al., 2019), future fMRI studies should implement value-imbalanced, between-category designs (e.g., healthy-Go vs. unhealthy-No-Go) to test whether the present CPM networks generalize to promoting less-preferred/healthier options. Sixth, our cue format (green frame for Go; gray frame for No-Go) may have modulated salience or affect; harmonized, cross-lab comparisons of cue properties are needed to quantify any impact on training efficacy. Seventh, choices in the in-scanner probe were not incentive-compatible—the selected item was not delivered for consumption—which may attenuate or distort revealed preferences relative to real consumption; future studies should incorporate random-lottery incentive delivery after scanning. The stimuli consisted exclusively of candy images. The findings therefore concern immediate choices among the specific candy stimuli used here and should not be generalized automatically to other foods or eating behavior. Eighth, we did not systematically screen for neurological disorders; the absence of a structured neurological-history assessment may constrain generalizability and introduce unmeasured confounds. Future work should include explicit neurological screening (e.g., a standardized checklist). Finally, the study was not preregistered and lacked an a priori power analysis, increasing risks of bias and imprecision; future investigations should preregister hypotheses/analysis plans and include formal power calculations.

## 5. Conclusions

In summary, using CPM in a substantial imaging sample, we found that task-evoked Go − No-Go functional connectivity modestly predicted individual differences in post-training Go-choice bias. MF–Mot, FP–Mot, and within-DMN connections were prominent in the positive predictive network, whereas MF–SAL connections formed a prominent component of the distributed negative network. These connections identify candidate predictive patterns compatible with possible contributions of action selection, internally represented goals and associations, and motivational salience. Independent replication and designs that directly measure these processes are required before the patterns can be considered generalizable neural signatures or clinically actionable predictors.

## Figures and Tables

**Table 1:** Ten nodes with the most connections contributing to the positive model.

Number	k	Node Name	Lobe	Network	BA	MNI Coordinates (x, y, z)		
Positive network								
1	8	Precentral gyrus	Motor strip	Motor	6	26.32	−12.91	66.20
2	8	Insula	Parietal	Motor	1	43.34	−10.81	13.91
3	8	Medial orbitofrontal gyrus	Prefrontal	Default Mode	10	−6.93	48.31	−5.71
4	8	Middle occipital gyrus	Occipital	Default Mode	19	−41.25	−75.44	22.76
5	7	Caudate	Subcortical	Subcortical	NA	12.56	20.19	−0.70
6	7	Inferior frontal gyrus	Prefrontal	Medial Frontal	47	−46.23	28.19	−7.12
7	7	Middle cingulate cortex	Motor strip	Motor	6	−6.47	−4.31	47.60
8	7	Middle cingulate cortex	Limbic	Motor	6	−7.75	−22.37	46.05
9	6	Middle orbitofrontal gyrus	Prefrontal	Frontal-parietal	10	30.51	54.92	−3.52
10	6	Middle frontal gyrus	Prefrontal	Frontal-parietal	10	28.88	51.14	18.68

k = number of stable connections incident to a node; BA = Brodmann area; NA = not applicable. Anatomical names indicate AAL labels at parcel centroids; lobe and BA labels follow the atlas annotations and may describe different parts of a parcel. Degree ties were ordered by Shen node number.

**Table 2:** Ten nodes with the most connections contributing to the negative model.

Number	k	Node Name	Lobe	Network	BA	MNI Coordinates (x, y, z)		
Negative network								
1	17	Inferior occipital gyrus	Occipital	Visual II	18	31.17	−91.77	−10.82
2	11	Calcarine	Occipital	Visual I	17	14.64	−68.33	8.28
3	11	Middle occipital gyrus	Occipital	Default Mode	19	−41.25	−75.44	22.76
4	10	Crus II of cerebellum	Cerebellum	Frontal-parietal	NA	11.72	−84.13	−34.65
5	10	Inferior occipital gyrus	Occipital	Visual II	18	−22.27	−96.64	−10.06
6	9	Calcarine	Limbic	Default Mode	23	12.34	−57.20	18.11
7	9	Supplementary motor area	Prefrontal	Medial Frontal	8	−5.03	17.67	46.05
8	9	Middle temporal gyrus	Temporal	Medial Frontal	21	−57.83	−47.48	5.24
9	9	Middle cingulate cortex	Limbic	Salience	31	−8.79	−42.55	50.11
10	8	Precentral gyrus	Motor strip	Salience	6	32.05	−5.36	52.05

k = number of stable connections incident to a node; BA = Brodmann area; NA = not applicable. Anatomical names indicate AAL labels at parcel centroids; lobe and BA labels follow the atlas annotations and may describe different parts of a parcel. Degree ties were ordered by Shen node number.

## Data Availability

The data presented in this study are available from the corresponding author upon reasonable request. The data are not publicly available because of privacy and ethical restrictions.

## Supplementary Materials

The following supporting information can be downloaded at: https://www.mdpi.com/article/10.3390/bs16101799/s1, Figure S1: Comparison of fixed edge-selection thresholds; Figure S2: CPM sensitivity analyses excluding participants with extreme choice-condition imbalance; Figure S3: CPM sensitivity analysis excluding participants with BMI ≥ 25 kg/m^2^ (*N* = 153); Table S1: Predictive performance across 100 repetitions; Table S2: CPM performance across five trial-matched draws (*N* = 162); Table S3: CPM performance using Go-condition connectivity (*N* = 162).
