# Supplementary material for: Task-Evoked Whole-Brain Functional Connectivity Predicts Post-Training Go-Choice Bias After Food Go/No-Go Training
Source: Behav Sci (Basel). 2026 Oct 2;16(10):1799. doi: 10.3390/bs16101799 (PMC13701987; doi:10.3390/bs16101799)
Supplement: Supplementary file 1 [file behavsci-16-01799-s001.zip › behavsci-4549983-supplementary.pdf]

Supplementary Materials

Task-Evoked Whole-Brain Functional Connectivity Predicts Post-Training Go-Choice Bias after Food Go/No-Go Training

Yingkai Yang

S1. Comparison of Fixed Edge-Selection Thresholds

Six fixed p-value thresholds (0.05, 0.01, 0.005, 0.001, 0.0005, and 0.0001) were evaluated in separate CPM runs, each using the same 100 repetitions of 10-fold cross-validation. Feature screening and model fitting used the training folds only. The mean combined-network Spearman correlations were 0.408, 0.380, 0.381, 0.238, 0.154, and 0.077, respectively. The $p < 0.05$ run had the highest observed mean correlation. The main analysis used $p < 0.05$. The threshold comparison was not nested within an additional outer cross-validation loop. CPM aggregates information across selected edges (Shen et al., 2017; Suo et al., 2022).

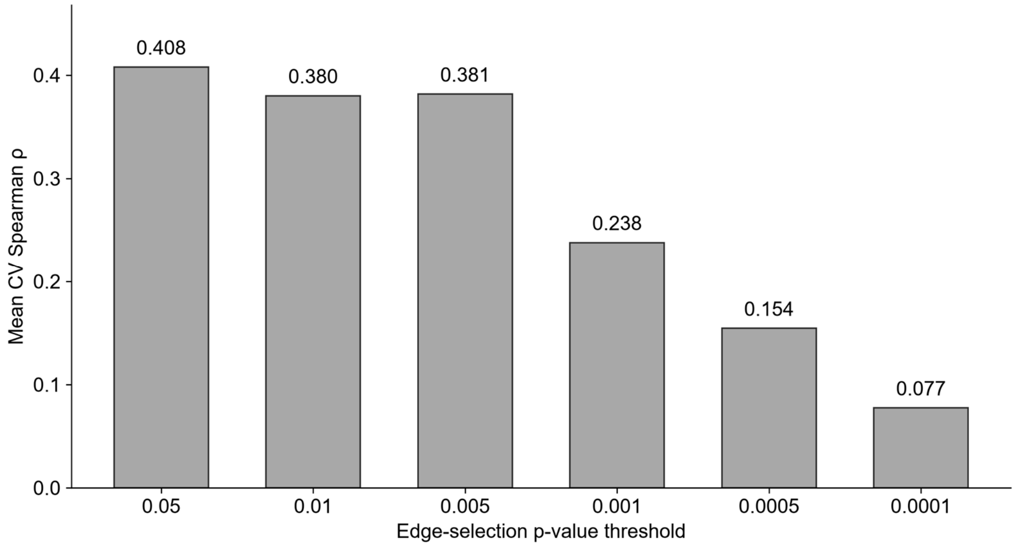

Figure S1. Comparison of fixed edge-selection thresholds. Values are the mean Spearman correlations across 100 repetitions for each separately run combined-network model.

S2. Exploratory Prediction of BMI

An exploratory CPM analysis used the same standardized Go − No-Go connectivity inputs to predict BMI, controlling for age, framewise displacement, and hunger at the feature-screening stage. Significance was assessed with 1,000 outcome permutations, each evaluated using the same single 10-fold cross-validation partition and Pearson correlation. Permutation p values were 0.760, 0.406, and 0.915 for the combined, positive, and negative networks, respectively. These results provide no evidence that the connectivity features predicted BMI in this sample under this procedure; they do not establish that BMI cannot contribute to the neural–behavioral association or that BMI-related confounding has been excluded.

S3. Variability Across Repeated Cross-Validation

Table S1 summarizes Spearman ρ and RMSE across 100 repetitions of 10-fold cross-validation at $p < 0.05$. Within each repetition, performance was calculated from observed and predicted Go-choice proportions for the 162 participants, with each participant evaluated in a test fold. The 25th–75th percentile intervals summarize variability across cross-validation partitions. Quartiles were calculated using linear interpolation. RMSE is expressed in Go-choice proportion units.

Table S1. Predictive performance across 100 repetitions.

Network	Metric	Mean	SD	Median	25th %ile	75th %ile
Combined	Spearman ρ	0.4077	0.0287	0.4062	0.3901	0.4265
Combined	RMSE	0.1691	0.0015	0.1692	0.1681	0.1701
Positive	Spearman ρ	0.3249	0.0371	0.3217	0.3024	0.3542
Positive	RMSE	0.1741	0.0019	0.1742	0.1728	0.1751
Negative	Spearman ρ	0.3937	0.0287	0.3949	0.3744	0.4121
Negative	RMSE	0.1687	0.0019	0.1685	0.1673	0.1698

S4. Sensitivity to Choice-Condition Imbalance

Two sensitivity analyses assessed the influence of pronounced imbalance between Go- and No-Go-choice trial counts. Requiring at least two valid choices in each condition excluded four participants ($N = 158$); requiring at least five excluded eight participants in total ($N = 154$). Both analyses retained the main model's connectivity inputs, covariates, and fixed edge-selection threshold ($p < 0.05$). Each analysis used 10-fold cross-validation repeated 100 times. Significance was assessed by permuting the Go-choice proportion 1,000 times and repeating the full 100-repeat procedure for each permutation, using the same partitions for the observed and permuted outcomes. The test statistic was the mean Spearman correlation across repetitions.

After excluding participants with fewer than two valid choices in either condition ($N = 158$), mean Spearman correlations were 0.386, 0.348, and 0.343 for the combined, positive, and negative networks, respectively (permutation $p < 0.001$, $p < 0.001$, $p = 0.002$). When at least five choices in each condition were required ($N = 154$), mean Spearman correlations were 0.270, 0.280, and 0.195 for the combined, positive, and negative networks, respectively (permutation $p = 0.005$, $p = 0.003$, $p = 0.023$). Predictive associations remained positive and significant across all three networks in both sensitivity analyses (Figure S2). These exclusions address extreme imbalance but do not equalize trial counts between conditions.

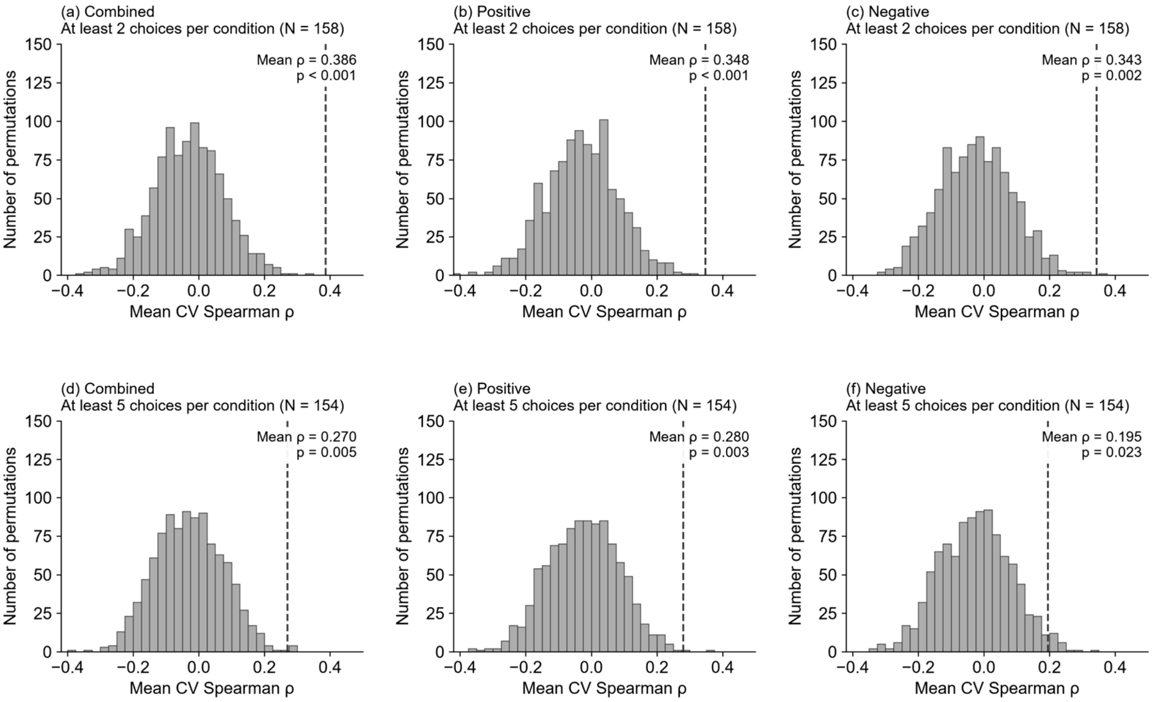

Figure S2. CPM sensitivity analyses excluding participants with extreme choice-condition imbalance. Panels (a–c) show the combined, positive, and negative networks for participants with at least two choices in each condition (N = 158); panels (d–f) show the corresponding networks for participants with at least five choices in each condition (N = 154). Gray histograms represent null distributions of mean Spearman ϱ across 100 repetitions of 10-fold cross-validation, obtained from 1,000 permutations of the behavioral outcome. Dashed lines indicate the observed mean Spearman ϱ. Feature selection and model fitting were repeated within each training fold for every permutation. Upper-tail permutation p values were calculated as (b + 1)/1,001, where b is the number of permuted statistics greater than or equal to the observed statistic. P values are uncorrected for comparisons across networks.

S5. Sensitivity to Exclusion of Participants with BMI ≥ 25 kg/m^2

Participants with BMI ≥ 25 kg/m^2 were excluded to examine whether the predictive associations were retained in the remaining sample. Of the ten participants meeting this criterion in the behavioral sample, one was already absent from the imaging sample; the CPM sample therefore decreased from 162 to 153. This analysis was conducted separately from the exclusions based on choice-condition imbalance. The model retained age, head motion, BMI, and hunger as covariates at the feature-screening stage and used the fixed $p < 0.05$ edge-selection threshold. Observed and permuted outcomes were evaluated with the same 100 repetitions of 10-fold cross-validation, repeating feature screening and model fitting within every training fold.

After excluding participants with BMI ≥ 25 kg/m^2 (N = 153), mean Spearman correlations were 0.425, 0.311, and 0.430 for the combined, positive, and negative networks, respectively (all permutation $p < 0.001$). Mean RMSE values were 0.173, 0.179, and 0.170, respectively. Predictive associations remained positive and significant across all three networks (Figure S3).

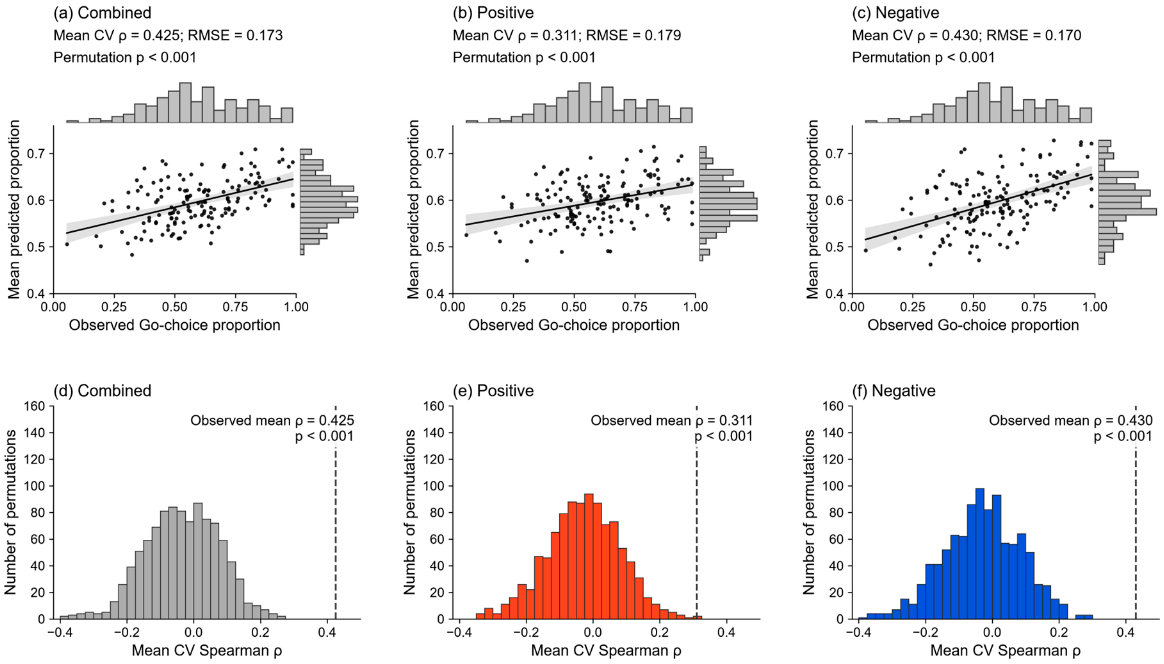

Figure S3. CPM sensitivity analysis excluding participants with BMI ≥ 25 kg/m^2 (N = 153). Panels (a–c) show observed Go-choice proportions and held-out predictions averaged across 100 repetitions for the combined, positive, and negative networks, with marginal histograms and descriptive linear fits with 95% confidence bands. Annotations report the mean within-repetition Spearman ϱ and RMSE. Panels (d–f) show null distributions of mean Spearman ϱ from 1,000 outcome permutations; each permutation repeated the full 100-repeat cross-validation procedure. Dashed lines indicate observed mean Spearman ϱ. All three upper-tail permutation p values were < 0.001, calculated as (b + 1)/1,001 without correction across networks.

S6. Sensitivity to Within-Participant Trial-Count Matching

Within each participant, the number of selected events in each condition was set to the smaller of the original Go-choice and No-Go-choice counts. All events from the smaller condition were retained, and events from the larger condition were randomly sampled without replacement. This procedure was repeated with five fixed random seeds (2026092701–2026092705), retaining all 162 participants and the original Go-choice proportion as the outcome. The gPPI model was re-estimated separately for each draw using the complete 232-volume time series. Selected Go and No-Go events and their unselected counterparts were modeled separately, including their main effects and PPI terms; unselected events were therefore retained in the model but did not enter the target contrast.

For each draw, the selected Go and No-Go connectivity matrices were symmetrized separately, subtracted, and standardized within participant across off-diagonal entries. CPM retained the main analysis's covariates and fixed edge-selection threshold ($p < 0.05$). Each draw used the same 100 repetitions of 10-fold cross-validation, with feature screening and model fitting confined to training folds. For each of 1,000 outcome permutations, the complete procedure was repeated using the same folds and the same permuted outcome across all five draws. The aggregate statistic was the mean Spearman ϱ across the five draws and 100 cross-validation repetitions, evaluated against the correspondingly averaged permutation statistics. Upper-tail p values were calculated as $(b + 1)/1{,}001$ and were uncorrected across the three networks.

Table S2. CPM performance across five trial-matched draws (N = 162).

Draw	Combined ρ	Combined p	Positive ρ	Positive p	Negative ρ	Negative p
1	0.1361	0.064	0.1416	0.068	0.0841	0.163
2	-0.0409	0.550	-0.0364	0.564	-0.0258	0.513
3	0.0147	0.344	0.1031	0.126	-0.0570	0.612
4	-0.0359	0.554	0.0972	0.132	-0.1247	0.829
5	-0.0686	0.652	-0.0234	0.501	-0.0713	0.670
**Aggregate**	**0.0011**	**0.361**	**0.0564**	**0.170**	**-0.0390**	**0.579**

Values are mean within-repetition Spearman correlations and upper-tail permutation p values. Aggregate statistics average all five draws; no draw was selected on the basis of performance.

Across the five draws, mean Spearman correlations were 0.001, 0.056, and −0.039 for the combined, positive, and negative networks, respectively (permutation p = 0.361, 0.170, and 0.579). No individual draw or aggregate network result reached $p < 0.05$ (Table S2).

Each draw assigned 7,598 of 11,368 valid events to the matched target conditions, a reduction of 33.2%; the remaining events were modeled separately. The number of selected events per condition ranged from 1 to 36 (median = 26), and eight participants contributed fewer than five events per condition. Thus, matching reduced the event information available to the target contrast and may have reduced estimation precision, particularly for participants with few minority-condition choices. The loss of significance cannot, however, be attributed to information loss alone: these results do not establish that the full-event association is independent of trial-count imbalance. No formal test of the difference between full-event and matched-event predictive performance was performed.

S7. Sensitivity Analysis Using Go-Condition Connectivity

A complementary sensitivity analysis predicted the original Go-choice proportion from Go-condition connectivity alone in all 162 participants. The Go-condition matrices were taken from the full-event gPPI model. The Go matrices were symmetrized and standardized within participant across off-diagonal entries. The analysis retained the main model's covariates, fixed $p < 0.05$ screening threshold, 100 repetitions of 10-fold cross-validation, and 1,000 outcome permutations, with the same folds and permutation orders. Feature selection and model fitting were repeated within every training fold.

Table S3. CPM performance using Go-condition connectivity (N = 162).

Network	Mean Spearman ρ	Mean RMSE	Permutation p
Combined	0.3142	0.1761	< 0.001
Positive	0.1704	0.1854	0.043
Negative	0.3655	0.1729	< 0.001

Mean Spearman correlations were 0.314, 0.170, and 0.365 for the combined, positive, and negative networks, respectively (permutation $p < 0.001$, $p = 0.043$, and $p < 0.001$; uncorrected across networks). Mean RMSE values were 0.176, 0.185, and 0.173, respectively (Table S3).

This analysis retains all Go events and shows that concurrent prediction can also be obtained without constructing a Go − No-Go connectivity difference.

References

Shen, X., Finn, E. S., Scheinost, D., Rosenberg, M. D., Chun, M. M., Papademetris, X., & Constable, R. T. (2017). Using connectome-based predictive modeling to predict individual behavior from brain connectivity. *Nature Protocols*, *12(3)*, 506-518. https://doi.org/10.1038/nprot.2016.178

Suo, X., Zuo, C., Lan, H., Pan, N., Zhang, X., Kemp, G. J., ... & Gong, Q. (2022). COVID-19 vicarious traumatization links functional connectome to general distress. *NeuroImage*, *255*, 119185. https://doi.org/10.1016/j.neuroimage.2022.119185
